# The effect of slope aspect on vegetation attributes in a mountainous dry valley, Southwest China

**DOI:** 10.1038/s41598-020-73496-0

**Published:** 2020-10-05

**Authors:** Jie Yang, Yousry A. El-Kassaby, Wenbin Guan

**Affiliations:** 1 School of Ecology and Nature Conservation, Beijing Forestry University, Beijing, China; 2 Department of Forest and Conservation Sciences, Faculty of Forestry, University of British Columbia, Vancouver, BC Canada

**Keywords:** Biodiversity, Community ecology, Plant sciences, Biodiversity, Community ecology

## Abstract

Slope aspect plays a critical role in influencing vegetation pattern in semiarid area. The dry valleys of the Hengduan Mountains Region, southwestern China, are striking geographical landscape, suffering from severe ecological degradation. Here, we comprehensively investigated how slope aspect affects vegetation attributes in one of these valleys- the dry valley in the upper reaches of Min River. Three sites were selected along the valley and we quantitively examined the vegetation difference between slope aspects at the whole valley scale and each site level. We found significant vegetation differences between slope aspects in species composition, vegetative structure, and biodiversity pattern, which were in accordance with the observed significant difference in soil nutrient. Generally, north-facing slopes are associated with higher biomass, coverage and height, and species diversity than south-facing slopes. We also found between-aspect differences varied among the study sites, resulting in increased biomass, height, and β diversity differences, decreased density and coverage differences, and opposite trend observed in α diversity at relatively wet site. In conclusion, slope aspect had significant effect on vegetation attributes, which was significantly influenced by local climate (aridity) in terms of both strength and direction depending on the specific attributes investigated.

## Introduction

Topographic elements, such as elevation, aspect, and position, significantly influence vegetation distribution and attributes through the modification of local environment^1–4^. Due to the different solar radiation received^5,6^, the differential environments and associated vegetation between slope aspects (i.e., equator- and pole-facing slopes), is a global phenomenon at middle latitude^5,7–9^. Especially in semiarid areas, slope aspect significantly influences microclimate (e.g., air and soil temperature, evapotranspiration, wind speed)^8,10,11^, soil property (e.g., organic matter content, chemical property, soil depth, soil texture)^12–15^ and hydrological processes (e.g., runoff dynamics, hydraulic conductivity, soil water retention)^16–19^, consequently resulting in distinct vegetation types occupying opposite slopes^5,8,20^. In general, the polar-facing slopes are wet and cool, with rich organic matter and deeper soil, and associated with mesic vegetation, while the equator-facing slopes are hot and dry, with low soil nutrient and severe soil erosion, and are occupied by xeric vegetation^8,20^.

Numerous studies have examined the effect of slope aspect on vegetation properties in semiarid areas, such as species composition and vegetation structure^5,8,21–24^. Generally, different species dominate these contrasting slopes and the polar-facing slope usually is associated with denser vegetation cover and higher productivity than their counterpart equator-facing slope^5,8,22^. However, the patterns of aspect-induced vegetation differences were not consistent among studies. On one hand, the strength of the effect of slope aspect on vegetation differed among study sites under semiarid climate. For example, Kutiel and Lavee^25^ found the soil and vegetation differences between north- and south-facing slopes in the semiarid and arid zones (rainfall < 400 mm) were small and generally negligible; however, Sternberg and Shoshany^26^ found significant vegetation difference between opposite slopes in semiarid regions and observed the strongest slope effects in areas with critical water limitation. On the other hand, different vegetation attributes showed different responses to the aspect-induced environmental variation. For example, Méndez-Toribio et al.^27^ found most vegetation structure attributes did not response to slope aspects while species diversity did show significant difference. Moreover, slope aspect could interact with other environmental variables, e.g. slope position, to jointly influence vegetation structure^27,28^. Furthermore, studies also found anthropogenic activity significantly increased differences in diversity pattern between opposite slopes^29^. Thus, a consistent conclusion and a universal generalization are still lacking about how slope aspect affects vegetation attributes in semiarid area. Additional empirical studies from natural ecosystems are required to obtaining a better understanding of the effect of slope aspect on vegetation.

The dry valleys of the Hengduan Mountains region, southwest China, are a striking geographical landscape, widely distribute in the main rivers and their tributaries in this region, notably along the upper Yangtze (Jinsha), Dadu, Yalong, Min, Lancang (Mekong), Nu (Salween), and Yuan (Red) and their tributaries^30^. These dry valleys are featured by much less rainfall, higher temperature and evaporation comparing to their neighboring areas, and are among the most fragile and degraded ecosystems in Southwest China. The dry valley in the upper reaches of Min River, the first-order branch of the Yangtze River, located on the transition zone from the Tibetan Plateau to the Sichuan Basin (30°44′–32°24′N, 102°41′–103°58′E), is one of these dry valleys. The high mountain-deep valley featured topography results in the strong foehn effect, giving rise to dry-warm climate with wet warm summer and dry cool winter. Soil erosion is severe, ecological degradation has been expanded due to both local climate, and human intervene. Vegetation belongs to winter drought scrubs mainly consisting of small-leaf arid shrubs^31^. Previous studies have examined the vegetation classification and ordination in environmental space^32^, spatial distribution of soil moisture^33^, differential response to soil variables between shrub and herb species^34^, and vegetation-soil-topography association^35^. Most of these studies focused on vegetation response to spatial variation in soil properties (nutrient and water); however, studies that precisely examine the topographic effect on vegetation attributes in this mountainous dry valley are lacking.

According to the geographical variation of the effect of slope aspect on vegetation property (which shows substantial strength occurred on steeper slopes at 30–45° N/S^5^), and the wide acknowledgement that both vegetation and soil were significantly influenced by topography^36^_ENREF_38, we hypothesize that slope aspect (north- vs. south-facing slope) has strong effect on vegetation attributes (focus on woody species) in this mountainous dry valleys through its modification on concomitant local environment. The objectives of this study were to: (1) examine and compare the soil properties between north- and south-facing slopes, (2) examine and compare species composition, vegetation structure, and biodiversity pattern between the opposite slopes, and (3) figure out the potential factors influencing the between-aspect vegetation difference (strength and direction). Three sites were selected along the Min River dry valley and the comparisons were conducted at three levels: between slope aspects in the whole valley, between slope aspects at each site and among the study sites along the valley. Specifically, several questions were addressed: (1) Do these vegetation attributes response in same way to the slope aspect? (2) Are the between-aspect differences in vegetation attributes similar among the three sites in terms of both strength and direction? (3) Are there other potential factors influencing vegetation difference except that of soil property? To the best of our knowledge, this is the first study to comprehensively examine the effect of slope aspect on vegetation attributes in the dry valley of the upper reaches of Min River. We hope that the present study findings would assist in better understanding of vegetation distribution, and ultimately provide important implication for biodiversity management and ecosystem restoration for contemporary and future climatic conditions, and finally to inspire future studies on other dry valleys in Hengduan Mountains region.

## Results

In the 42 study plots, a total of 52 woody species belonging to 34 genera and 19 families were recorded, among which Fabaceae was the largest family containing 12 species accounting for 23% of total species.

### Soil property

Comparison of edaphic variables showed significant differences in soil nutrient (organic matter (ORG), available nitrogen (NA) and available potassium (KA)) between north- and south-facing slopes at the whole valley (ORG_.north_ = 14.82 ± 1.88%, ORG_.south_ = 6.16 ± 0.45%, *p* = 6.81E−06; NA_.north_ = 361.45 ± 35.91 mg kg^−1^, NA_.south_ = 181.38 ± 13.39 mg kg^−1^, *p* = 8.47E−07; KA_.north_ = 178.65 ± 8.54 mg kg^−1^, KA_.south_ = 107.33 ± 4.26 mg kg^−1^, *p* = 3.13E − 10) and at all three study sites levels (Table 1). In contrast, no significant difference in soil moisture (SM) between slope aspects was found at the whole valley (SM_.north_ = 10.28 ± 0.88%, SM_.south_ = 8.73 ± 0.78%, *p* = 0.124) and at study sites except for Feihong where the SM was significantly higher on north-facing slope (*p* = 0.006) (Table 1). Moreover, the among-site comparison showed significantly higher SM at Shidaguan than Wenchuan and Feihong (SM_.Shidaguan_ = 13.22 ± 2.46%, SM_.Wenchuan_ = 6.41 ± 1.59%, SM_.Feihong_ = 7.21 ± 2.14%, *p* = 5.13E − 11) (Table 1).

**Table 1:** Results of one-way ANOVA comparison of numerical environmental variables between slope aspects for all studied sites.

Parameter	Wenchuan		Feihong		Shidaguan	
	North (6)	South (7)	North (5)	South (7)	North (7)	South (10)
Elevation (m)	1428.33 (37.10)^a^	1437.86 (53.37)^a^	1931.00 (36.22)^a^	1798.57 (88.77)^b^	2047.14 (82.00)^a^	2013.20 (51.63)^a^
Degree (°)	32.83 (5.04)^a^	40.00 (3.96)^b^	38.00 (3.46)^a^	36.57 (1.40)^a^	40.00 (0.58)^a^	32.45 (4.65)^b^
Soil moisture—SM (%)	6.77 (2.20)^a^	6.10 (0.88)^a^	9.00 (0.78)^a^	5.93 (1.85)^b^	14.20 (1.85)^a^	12.53 (2.69)^a^
pH	7.93 (0.12)^a^	7.93 (0.16)^a^	7.20 (0.35)^a^	7.39 (0.49)^a^	7.51 (0.27)^a^	7.44 (0.43)^a^
Organic matter content—ORG (%)	9.14 (3.66)^a^	4.88 (1.33)^b^	23.78 (9.61)^a^	5.85 (1.48)^b^	13.28 (2.29)^a^	7.27 (2.61)^b^
Available nitrogen—NA (mg kg^−1^)	260.87 (82.84)^a^	124.15 (28.20)^b^	526.45 (184.45)^a^	191.27 (48.92)^b^	329.79 (58.35)^a^	214.51 (70.88)^b^
Available phosphorus—PA (mg kg^−1^)	1.98 (2.05)^a^	0.81 (0.47)^a^	5.26 (2.75)^a^	6.95 (2.23)^a^	4.00 (1.12)^a^	3.55 (3.08)^a^
Available potassium—KA (mg kg^−1^)	188.38 (32.08)^a^	95.09 (14.72)^b^	161.02 (44.62)^a^	114.24 (18.68)^b^	182.90 (34.03)^a^	111.07 (23.76)^b^

Mean and standard deviation (SD) followed with different letters indicate significant differences at p < 0.05 between north and south aspects. Number of sampling plots within each slope is presented in brackets.

### Vegetation composition

**Figure 1:**
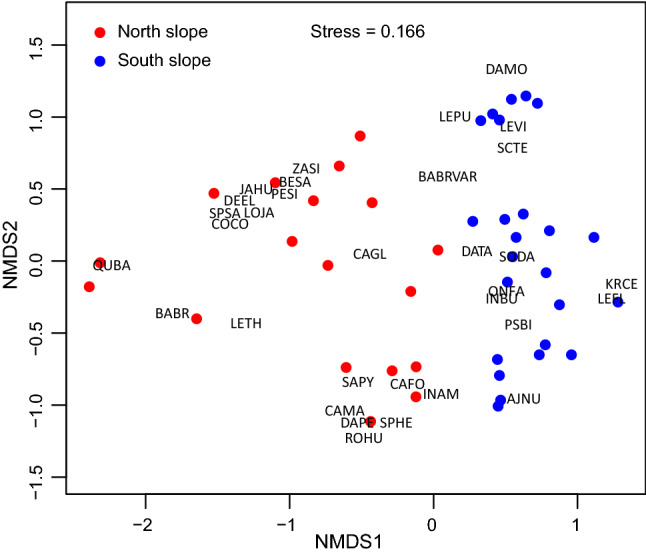
Nonmetric multidimensional scaling (NMDS) of 42 plots (north-facing slope N = 18, south-facing slope N = 24) based on Bray–Curtis dissimilarity (stress = 0.166). Plots located on different slope aspects were represented by different colors: red (north slope); blue (south slope). Species abbreviation: *Quercus baronii* (QUBA), *Bauhinia brachycarpa* (BABR), *Desmodium elegans* (DEEL), *Jasminum humile* (JAHU), *Spiraea salicifolia* (SPSA), *Lonicera japonica* (LOJA), *Cotinus coggygria* (COCO), *Pertya sinensis* (PESI), *Berberis sargentiana* (BESA), *Zanthoxylum simulans* (ZASI), *Sageretia pycnophylla* (SAPY), *Caryopteris forrestii* (CAFO), *Campylotropis_macrocarpa* (CAMA), *Daphne penicillate* (DAPE), *Rosa hugonis* (ROHU), *Spiraea henryi* (SPHE), *Indigofera amblyantha* (INAM), *Caryopteris terniflora* (CATE), *Sophora davidii* (SODA), *Bauhinia brachycarpa* var. *microphylla* (BABRVAR), *Onosma farreri* (ONFA), *Indigofera bungeana* (INBU), *Caryopteris bicolor* (CABI), *Leptodermis purdomii* (LEPU), *Daphne tangutica* (DATA), *Lespedeza virgate* (LEVI), *Ajania nubigena* (AJNU), *Lespedeza thunbergii* (LETH), *Lespedeza floribunda* (LEFL), *Caryopteris glutinosa* (CAGL), *Krascheninnikovia ceratoides* (KRCE), and *Daphne modesta* (DAMO).

Nonmetric multidimensional scaling (NMDC) showed woody species composition on north- and south-facing slopes were strongly separated in ordination space with no overlap (stress value = 0.166) (Fig. 1). The analysis of similarity (ANOSIM) showed significant compositional difference between slope aspects (R = 0.46, *p* = 0.001). Sorensen dissimilarity between aspects were 0.75, 0.77, and 0.89 for Shidaguan, Feihong, and Wenchuan, respectively, indicating considerable floristic difference between north- and south-facing slopes for all study sites.

### Vegetation structure

**Figure 2:**
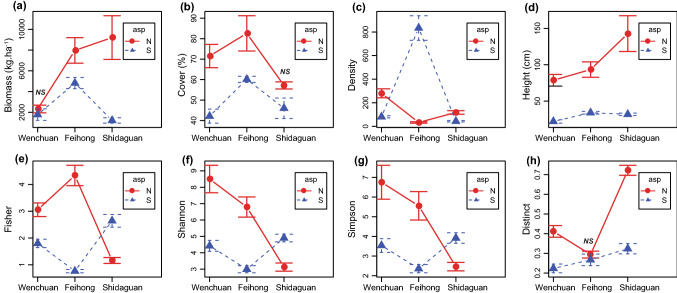
Comparison of vegetation structure **a** biomass, **b** coverage, **c** height, **d** density and biodiversity, **e** Fisher’s α, **f** Shannon diversity, **g** Simpson diversity, and **h** distinct between north- (red) and south-facing (blue) slopes for each site using one-way ANOVA.

Comparison of vegetation structure at the whole valley scale showed that structure attributes (biomass, coverage, and average height) were significantly higher on north- than south-facing slope (biomass_.north_ = 6567.90 ± 1124.89 kg ha^−1^, biomass_.south_ = 2430.49 ± 401.05 kg ha^−1^, *p* = 4.21E−04; cover_.north_ = 69.00 ± 3.85%, cover_.south_ = 49.00 ± 2.73%, *p* = 8.75E−05; height_.north_ = 107.72 ± 12.04 cm, height_.south_ = 29.58 ± 1.49 cm, *p* = 4.9E−09), and a similar pattern was observed at all study sites level (Table 2, Fig. 2a–d). In contrary, density was higher (but not significant) on south- than north-facing slope at the whole valley scale (density_.north_ = 148.39 ± 27.52, density_.south_ = 284.67 ± 79.06, *p* = 0.656) and with different patterns among sites, where the significantly higher density was on the south-facing slope at Feihong but on north-facing slopes at Shidaguan and Wenchuan (Table 2, Fig. 2e). The between-aspect vegetation differences varied among sites, with Shidaguan showing the highest difference in biomass and height and lowest difference in density and coverage as compared to the other two sites (Table 2, Fig. 2).

**Table 2:** Comparison of vegetative structure and biodiversity variables between slope aspects using one-way ANOVA for the whole valley scale and each site level (Mean ± standard error) values are shown, differ_.n-s_, difference value between north- and south-facing slopes).

Structure	Biomass (kg ha^−1^)				Cover (%)				Height (cm)				Density			
	North	South	Differ_n-s_	*p*	North	South	Differ_n-s_	*p*	North	South	Differ_n-s_	*p*	North	South	Differ_n-s_	*p*
Wenchuan	2322.7 (± 371.41)	1778.61 (± 554.94)	544.09	0.449	71.5 (± 5.67)	42.14 (± 3.43)	29.36	0.001	78.67 (± 7.96)	22.00 (± 2.35)	56.67	< 0.001	280.00 (± 38.39)	80.57 (± 8.98)	199.43	< 0.001
Feihong	7966.39 (± 1227.91)	4819.81 (± 536.61)	3146.58	0.026	82.6 (± 8.62)	60.14 (± 1.52)	22.46	0.012	93.40 (± 10.64)	34.00 (± 1.90)	59.40	< 0.001	33.00 (± 6.04)	833.86 (± 104.35)	− 800.86	< 0.001
Shidaguan	9207.72 (± 2105.67)	1214.30 (± 258.84)	7993.42	< 0.001	57.14 (± 1.74)	46.00 (± 4.99)	11.14	0.092	142.86 (± 24.64)	31.80 (± 1.69)	111.06	< 0.001	118.00 (± 15.26)	43.10 (± 5.80)	74.90	< 0.001
Total	6567.9 (± 1124.89)	2430.49 (± 401.05)	4137.41	< 0.001	69.00 (± 3.85)	49.00 (± 2.73)	20.00	< 0.001	107.72 (± 12.05)	29.58 (± 1.49)	78.14	< 0.001	148.39 (± 27.52)	284.67 (± 79.06)	− 136.28	0.656

Diversity	Fisher’s α				Shannon				Simpson				Distinct			
	North	South	Differ_n-s_	*p*	North	South	Differ_n-s_	*p*	North	South	Differ_n-s_	*p*	North	South	Differ_n-s_	*p*
Wenchuan	3.06 (± 0.25)	1.80 (± 0.16)	1.26	< 0.001	8.50 (± 0.84)	4.42 (± 0.33)	4.08	< 0.001	6.75 (± 0.86)	3.54 (± 0.35)	3.21	0.004	0.41 (± 0.03)	0.22 (± 0.02)	0.19	< 0.001
Feihong	4.33 (± 0.38)	0.77 (± 0.06)	3.56	< 0.001	6.79 (± 0.62)	2.99 (± 0.22)	3.80	< 0.001	5.55 (± 0.72)	2.36 (± 0.21)	3.19	< 0.001	0.29 (± 0.02)	0.27 (± 0.03)	0.02	0.896
Shidaguan	1.16 (± 0.11)	2.64 (± 0.24)	− 1.48	< 0.001	3.13 (± 0.25)	4.90 (± 0.23)	− 1.77	0.001	2.46 (± 0.21)	3.92 (± 0.27)	− 1.46	0.002	0.72 (± 0.03)	0.32 (± 0.03)	0.40	< 0.001
Total	2.68 (± 0.34)	1.85 (± 0.19)	0.83	< 0.001	5.94 (± 0.65)	4.21 (± 0.22)	1.73	0.006	4.75 (± 0.57)	3.35 (± 0.21)	1.40	0.013	0.50 (± 0.05)	0.28 (± 0.02)	0.22	< 0.001

### Biodiversity pattern

**Figure 3:**
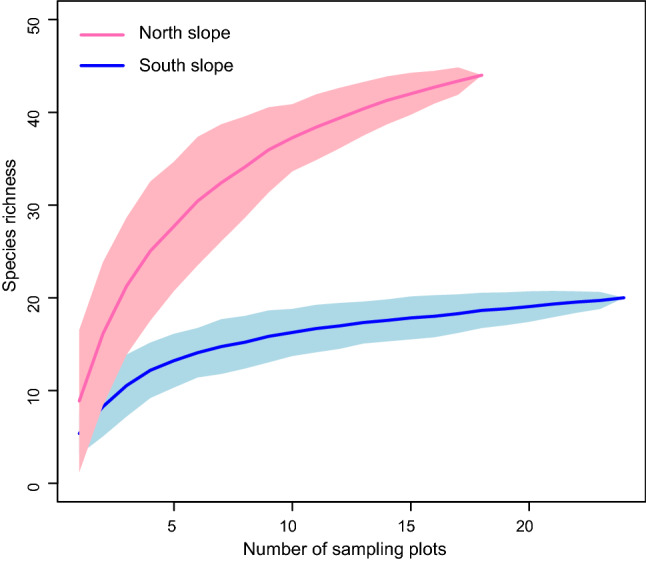
Gamma (γ) diversity with 95% confidence intervals for the north- (pink) and south-facing (blue) slopes based on sample-based rarefaction. North slope (N = 18), south slope (N = 24).

A total of 44 and 20 woody species were recorded on north- and south-facing slopes, respectively. Sample-based rarefaction showed significantly higher species richness (γ diversity) on north- than south-facing slope for all sampling efforts (Fig. 3). Both Chao2 and Jackknife2 showed higher estimation of species diversity on north-facing slope (Chao2_north_ = 63.95 ± 15.55 and Jackknife2_north_ = 64.49 ± 8.83 vs. Chao2_south_ = 31.98 ± 16.44 and Jackknife2_south_ = 28.50 ± 3.16).

For the whole valley scale, all α diversity metrics were significantly higher on north-facing slope (Fisher_north_ = 2.68 ± 0.34, Fisher_south_ = 1.85 ± 0.19, *p* = 0.002; Shannon_north_ = 5.94 ± 0.65, Shannon_south_ = 4.21 ± 0.22, *p* = 0.006; Simpson_north_ = 4.75 ± 0.57, Simpson_south_ = 3.35 ± 0.21, *p* = 0.013); however, the pattern at Shidaguan was inconsistent with the general pattern and other sites where all α diversity metrics were significantly higher on south-facing slope (Table 2, Fig. 2e–g).

**Figure 4:**
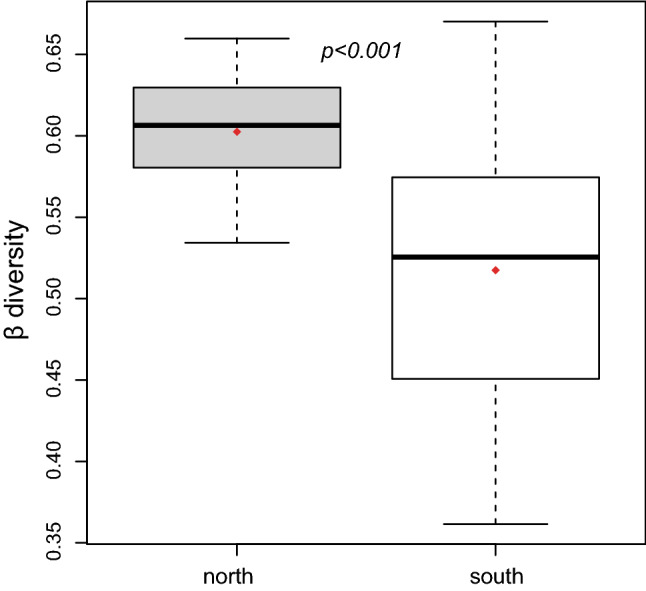
Comparison of floristic heterogeneity (β diversity) using distance-to-centroid (d_cen_) between north- (gray) and south-facing (white) slopes by one-way ANOVA.

Comparison of local floristic distinctiveness (Distinct) showed significantly higher value on north-facing slope at the whole valley scale (Distinct_north_ = 0.50 ± 0.05, Distinct_south_ = 0.28 ± 0.02, *p* = 1.06E−05) but not Feihong, where no significant difference was found between the opposite slopes (*p* = 0.896) (Table 2, Fig. 2h). At landscape scale, the multivariate woody β diversity was significantly higher on north- than south-facing slope (β_north_ = 0.603 ± 0.036, β_south_ = 0.517 ± 0.080, *p* = 1.38E−04) (Fig. 4), indicating significantly decrease of vegetative heterogeneity on the south-facing slopes.

## Discussion

Our study demonstrated significant differences in species composition, vegetative structure, and biodiversity pattern between north- and south-facing slopes in the mountainous dry valley of the Min River. Generally, north-facing slopes were associated with higher biomass, coverage, height, species diversity than south-facing slopes; this was in accordance with the higher soil nutrient content on north-facing slopes. Moreover, depending on the specific vegetation attribute of interest, the between-aspect vegetation differences varied among sites in terms of both strength and direction, indicating the presence of other factors that might influence the effect of slope aspect on vegetation.

Soil water limitation and nutrient deficiency are dominant environmental constraints for vegetation distribution in semiarid area, which are greatly influenced by topography^37^. We found significantly higher soil nutrient content (available nitrogen (NA), organic matter (ORG), and available potassium (KA)) on the north-facing slopes at the whole valley scale and all three study sites level (Table 1), results are consistent with the observed patterns of other semiarid ecosystems, such as the Chinese Loess^13^ and Mediterranean regions^14,25^. Contrary to our expectation and previous semiarid area studies^16,17,19^, no significant difference in soil moisture between slope aspects was found at neither the whole valley scale nor the study site level except for Feihong which showed significantly higher soil moisture on the north- than south-facing slope (Table 1). We surmised that this incongruence might occur as soil moisture measurement in this study was conducted in sunny days with absence of rainfall event. In fact, soil moisture tends to vary temporally and the pulse of soil water after rainfall event is critical for plant growth in semiarid area^38,39^. Several studies have showed a significantly larger soil water storage on north-facing slope^12^ and that the between-aspect difference in soil water decreased with time after rainfall^25^. Therefore, the soil moisture measurement in our study might have underestimated the difference between-aspect. Nevertheless, soil nutrient and moisture were tightly correlated. On one hand, high soil moisture increases the positive feedback between productivity and soil fertility^40^_ENREF_41 and accelerates the microbial activities for nitrogen-mineralization^41,42^, resulting in more organic matter content and nitrogen availability, while on the other, the higher organic matter content increases soil water retention through the improvement of soil structure which determined the water infiltration and maintenance^37^. Thus, the observed significant difference in soil nutrient (e.g., NA and ORG) might reflect different soil moisture between slope aspects (Table 1). Moreover, the among-site comparison showed Shidaguan had significantly higher soil moisture (2 times more) than Wenchuan and Feihong (p < 0.001), confirming the significant aridity difference between Shidaguan (wet local climate) and the other two sites (dry local climate)^43^.

The observed strong differentiation between north- and south-facing slope vegetation compositions is probably due to the different ecological adaptation of plant species on these contrasting slopes. The mesic north-facing slopes were occupied by drought-avoiding species (*Quercus baronii*, *Berberis sargentiana*, *Cotinus coggygria*, *Jasminum humile*, *Campylotropis macrocarpa*, and *Daphne penicillata*), while the harsh xeric south slopes were dominated by drought-tolerant species and resistant to nutrient deficiency (*Caryopteris bicolor*, *Caryopteris terniflora*, *Sophora davidii*, *Bauhinia brachycarpa* var. *microphylla*, *Indigofera bungeana*, and *Lespedeza virgate*). It is worth noting that abundant *Ajania nubigena* dominated the south-facing slope of Feihong, which had the harshest growing environment that is characterized by moisture and nutrient lacking soil (Table 1). This strong separation of species composition between opposite slopes supports Guan et al.^32^ vegetation classification, which identified distinct vegetation formations associated with different slope aspects. For example, Fabaceae represented the largest family in this dry valley, especially with *Sophora davidii* and *Bauhinia brachycarpa* var. *microphylla* as the dominant species with their unique drought-tolerant, nitrogen fixation, and water and soil conservation attributes^44–46^. Additionally, drought-tolerant species such as *Ajania nubigena* and *Caryopteris* spp. dominated the south-facing slopes at Wenchuan and Feihong sites characterized by their soil water and nutrient deficiency^32,47^. Therefore, the difference in soil water and nutrient between slope aspects might act as a predominant environmental filtering of species survival on opposite slopes. Notably, besides edaphic property, slope aspect could also affect vegetation distribution through its effect on microclimate, particularly air and soil temperature, which affect plant establishment and growth^48,49^. Our results are consistent with other semiarid area worldwide studies that showed coexistence of aspect-delimited ecosystems in very close proximity^22,50^.

Vegetation structure showed significant difference between north- and south-facing slopes with all biomass, cover and height attributes being significantly higher on north-facing slope (Table 2). Similar pattern was also found in other studies in semiarid areas that indicated the existence of favorable environmental conditions (higher soil nutrient and water) on north-facing slopes, thus benefiting plant growth^5,25^. Most notably, the slope aspects comparison across sites showed vegetation difference varied among sites and the trend of variation differed among vegetation structure variables (Table 2). For example, the difference in biomass and height between slope aspects were much higher while the difference in coverage and density were much lower at Shidaguan than Feihong and Wenchuan (Table 2, Fig. 2), indicating that there must be some factors influencing the effect of slope aspect on vegetation structure. Shidaguan, which is located at the northern end of the dry Min River valley, has relatively wet local climate as compared to Wenchuan and Feihong^43^, thus its mesic environment could have facilitated plant growth and consequently resulted in higher vegetation difference between aspects (Table 1). In contrast, the dry local climate at Feihong and Wenchuan caused severe water limitation which restricts plant growth on both slope aspects and consequently could diminish the vegetation difference between slope aspects. Similar pattern was also reported by Fernandez-Going et al.^40^, demonstrating the functional dissimilarities between communities on infertile serpentine and fertile non-serpentine soils were higher in more productive (wetter) regions in California. However, in contrary to the height and biomass, coverage and density (usually positively correlated) showed smaller differences between slope aspects at Shidaguan than Wenchuan and Feihong. This was mainly because of the presence of *Quercus baronii*, the dominant species on the north-facing slope at Shidaguan, which had large individual size with average height more than 200 cm and high species biomass^51^. Besides, the associated species such as *Bauhinia brachycarpa* var. *microphylla* and *Lespedeza thunbergii* also showed relatively larger individual sizes than those growing at the other two sites. Consequently, the large individual plant size resulted in the decreased density and coverage on north-facing slope which were getting close to those on south-facing slopes. Analogously, the significantly higher density on south-facing slope at Feihong was due to the great abundance of the dominant species, *Ajania nubigena*, which not only increased the community density (contributing about 60% to community density) but also increased community biomass (contributing about 50% to community biomass) and coverage (Table 2, Fig. 2). Consequently, Fisher’s α diversity was lowest on the south-facing slope at Feihong, reflecting the highly unbalanced species abundance distribution (Table 2, Fig. 2). Therefore, the characters of the constituent species, especially the dominant species, substantially influenced the specific vegetation structure variable and consequently the difference between slope aspects vegetation.

All the α, β and γ diversity were significantly higher on north- than south- facing slope for the whole valley scale and at all sites level except for Shidaguan where the significantly higher α diversity occurred on south-facing slope (Table 2). Analogous to lower community density and coverage, the dominance of the competitive *Quercus baronii* on north-facing slope at Shidaguan also decreased α diversity through the competitive exclusion of occurring species in the favorable environment^52^. Similar pattern was also detected by Badano et al*.*^22^ who found higher species richness on xeric than on mesic slopes due to the decrease in importance of negative interactions from mesic to xeric habitats. In contrast, α diversity was higher on north-facing slope at Wenchuan and Feihong, result was consistent with previous observations in semiarid areas at middle latitude^5^. At landscape scale, both interpolative and extrapolative estimation of species diversity showed higher γ diversity on north- than south-facing slope, this might be due to the higher productivity (biomass) on north-facing slopes (favorable environment with higher soil nutrient and moisture) supporting the positive species-productivity relationship at regional scale^53^. β diversity at both plot and landscape scale were higher on north-facing slope, and the results were consistent with that reported in arid trans-Himalaya mountainous area^29^ and seasonal tropical dry forest^28^. We attributed this to the higher environmental heterogeneity on north-facing slope, which generates a wider diversity of microhabitats for species colonization, supporting the positive relationship between environmental heterogeneity and spatial variation in species composition^54–56^. This was well reflected in the higher variance of edaphic variables in north aspect (Table 1). Moreover, we found significant effect of slope configuration on plot distinctiveness (Distinct_concave_ = 0.57 ± 0.21, Distinct_flat_ = 0.30 ± 0.08, Distinct_convex_ = 0.25 ± 0.08, *p* = 1.17E−06) with much more concave configurations were observed on north- than south-facing slope, which might also contribute to the higher β diversity on north aspect. This study firstly investigated the effect of slope aspect on β diversity in this area, which provided better understanding of diversity component, for example, although Shidaguan had the lowest α diversity it simultaneously had the highest β diversity, indicating the joint effect of competition and environmental heterogeneity on diversity pattern.

As the first comprehensive study investigating how slope aspect affects vegetation attributes in the dry valley of the upper reaches of Min River, we found slope aspect significantly affects species composition, vegetation structure, and diversity pattern and the results were in accordance with the observed significant difference in soil nutrient between slope aspects. We also found the effect of slope aspect on vegetation was significantly influenced by local climate (aridity) in terms of both strength and direction depending on the specific vegetation attribute investigated. The present study represents the start of a comprehensive research program intended to inspire future studies related to the effect of topography on vegetation of the dry valleys in Hengduan Mountains region. We believe that the obtained results will be instrumental in providing valuable and insightful information on ecosystem restoration and biodiversity conservation under climate change.

## Method

### Study area

The study area is located in the central part of the dry-warm valley in the upper reaches of the Min River (alt. 1300–2200 m; lat. 30°44′–32°24′N; long. 102°41′–103°58′E), covering the Wenchuan, Maoxian, and Lixian counties in Sichuan province, Southeast China. This location has mean annual temperature of 11.2*–*12.9 °C, mean annual precipitation of 409–462 mm (with 70–80% occurring throughout May–October), and mean annual evaporation of 1300–1800 mm^57^. The soil is classified as Calcic cambisols (FAO-UNESCO, 1988) with shallow depths (10–30 cm) and coarse texture^30^. Sparse vegetation distributed in a mosaic of vegetated patches, isolated plants, and bare surfaces, which was dominated by drought-tolerant shrub species, such as *Sophora viciifolia*, *Bauhinia faberi* var. *microphylla* and *Indigofera bungeana*, and accompanied by herbaceous species, such as *Ajania breviloba*, *Sedum wenchuanense* and *Heteropogon contortus*^30^.

### Data collection

This study is based on field investigation dataset collected during August 2006 along the central part of the Min River dry valley. Three sites were selected along the valley representing different vegetation types: (1) Shidaguan at the upper end was dominated by short dry shrubs and grasses on both north- and south-facing slopes; (2) Feihong at the middle mainly consisted of sparse dry dwarf shrubs on north-facing slopes and sparse grasses on south-facing slopes; and (3) Wenchuan at the southern end had some small trees growing on north-facing slopes but only sparse shrubs on south-facing slopes (Supplementary Fig. S1 online). Moreover, the aridity extent has significant differentiation among the sites, where Wenchuan and Feihong being more arid than Shidaguan^43^. At each site, forming a V shape, two transects were set along north- and south-facing slopes. Sample plots of 5 × 5m^2^ were set up along the transects at altitude intervals of about 20 m. In each plot, all woody species were identified and measured of abundance, coverage (%), and height (cm). Above-ground biomass of each woody species in each plot were measured following the methods of Liu et al*.*^51^. A total of 42 plots formed the study dataset, with 18 and 24 plots located on north- and south-facing slopes, respectively (see Table 1 for the number of sampling plots on each site). Species nomenclature followed the Flora of China (https://foc.efora.cn/).

For each sampling plot, a total of 11 environmental variables were measured including topographical characteristics: elevation (*ele*—m), slope degree (*deg*—°), shape (*shp*—concave, flat or convex), position (*pos*—upper, middle or bottom of the slope) and aspect (*asp*—north or south), and edaphic properties: soil moisture (*SM*—%), organic content (*ORG*—%), available nitrogen *(NA*—mg kg^−1^), available potassium (*KA*—mg kg^−1^), available phosphorus (*PA*—mg kg^−1^), and pH. The elevation and slope degree were measured using an altitude meter and a clinometer, respectively. Within each sampling plot and on sunny days within a 2-week period without rainfall, soil moisture content was measured from the surface soil (0–15 cm) at nine points using a portable Time Domain Reflectometry (TDR)^34^. For soil chemical analyses, five surface soil samples (0–15 cm) were collected using cores (5 cm diameter) from five random soil profiles of each plot, air-dried, thoroughly mixed and passed through a 2 mm sieve to remove gravel and debris (see Lu et al.^34^, for details on lab analyses of soil chemical properties)_ENREF_34.

### Vegetation composition

Nonmetric multidimensional scaling (NMDS)^58^ was performed to visualize the woody compositional variation between north- and south-facing slopes based on the importance value (I.V., sum of the relative abundance, coverage and height of each species) of the most abundant species (occurrence frequency > 5%, i.e. occupation > 2 plots). Analysis of similarity (ANOSIM)^59^, a non-parametric multivariate analysis, was used to test the overall compositional difference between north- and south-facing slopes based on Hellinger distance (permutation 999 times). Sorensen dissimilarity between opposite slopes was also calculated for each site. All analyses were performed using R package ‘vegan’^60^.

### Vegetation structure

Biomass (kg ha^−1^), coverage (%), density, and average height (cm) were used as measures of vegetation structure. Above-ground biomass of each plot was estimated by the sum of above-ground biomass of all constituent woody species weighted by their relative abundance. Woody species biomass was used as community biomass as it accounted for more than 80% of total community biomass^61^. Coverage was the visual estimate of percentage canopy cover in each plot. Density was the total number of individual woody plants per plot. Plot average height was the mean species height weighted by their relative abundances.

### Vegetation diversity

We examined the diversity differences between north- and south-facing slopes in terms of different diversity aspects: (1) γ diversity was the species richness at respective north- and south-facing slopes of the whole valley; (2) α diversity was the species diversity measurement at sampling plot level; and (3) β diversity was the composition variation among sampling plots.

### γ diversity

Sample-based rarefaction curves with 95% confidence intervals was used to compare differences in species richness (γ diversity) between north- and south-facing slopes with different sample sizes using a Monte Carlo randomization procedure^62^. Chao2^63^ and Jack2^64^, nonparametric incidence-based species richness estimators, were also used to estimate species richness in a species pool for north- and south-facing slopes, respectively, taking into account both the number of species found in one sample only and in precisely two samples.

### α diversity

Species abundance data was used for α diversity analysis. Fisher’s α diversity, Shannon index, and Simpson index were used as α diversity metrics^65^. Fisher’s α is a parameter of Fisher’s log series model^66^, which has been recommended as a sample-size-independent estimator of richness predicting the number of species represented by a single individual^65^. For Shannon index, $D_{Shannon} = - \sum\nolimits_{1}^{S} {p_{i} \ln p_{i} }$, where *S* is the number of species, *p*_*i*_ is the proportion of individuals found in the *i*th species (*p*_*i*_ = *n*_*i*_/*N*), *n*_*i*_ is the number of individuals of species *i* in the sample, and N is the total number of individuals sampled. For Simpson index, $D_{Simpson} = 1/\sum\nolimits_{1}^{S} {p_{i}^{2} }$, symbols similar to those of Shannon index. The Shannon index is sensitive to changes in the proportions of rare species, while Simpson index is sensitive to changes in the proportions of common species. All the metrics were calculated using R package ‘vegan’^60^.

### β diversity

β diversity was estimated at the local (plot) and landscape levels. Firstly, the floristic distinctness of each plot (Distinct) from its surrounding vegetation was estimated by calculating the average pairwise Sorensen dissimilarity^67^ between the focal plot and all the other plots within the same transect. This estimate is a variation of β diversity measurement of grid system^68^ according to the sampling design in the present study and allowed the comparison between slope aspects using classical statistic method. At the landscape scale, floristic heterogeneity for north- and south-facing hillslopes of the whole valley were estimated using the distance-to-centroid (d_cen_), a multivariate β diversity metric which measured the average dissimilarity from individual plots to their group centroid^69,70^. This metric was proposed by Anderson^69^ to estimate the homogeneity of multivariate dispersion and had the advantage to allow β diversity comparison among different areas or groups of samples. The pairwise dissimilarity was estimated by Bray–Curtis dissimilarity using function ‘vegdist’ and distance-to-centroid using function ‘betadisper’ in R package ‘vegan’^60^.

### Statistical analysis

Vegetation structure, biodiversity (α diversity and plot Distinct), and soil variables between north- and south-facing slopes comparisons were conducted for the whole valley and at each site using one-way analysis of variance (ANOVA). To meet the assumptions of normality and homoscedasticity, data transformation was conducted: percentage data (i.e., coverage, SM and ORG) were arcsine transformed and count data (density) were square root transformed. If the parametric assumptions were not met, Kruskal–Wallis rank analysis of variance was used. The multivariate β diversity d_cen_ comparison between north- and south-facing slopes for the whole valley was conducted using one-way ANOVA. All analyses were conducted in R^71^, and statistical significance was set at *p* < 0.05 (two-tailed)_ENREF_73.

### Data availability statement

The datasets analysed during the current study are available from the corresponding author on reasonable request.

## Supplementary information

Supplementary Figure S1.
