# Supplementary material for: The effect of slope aspect on vegetation attributes in a mountainous dry valley, Southwest China
Source: Sci Rep. 2020 Oct 5;10:16465. doi: 10.1038/s41598-020-73496-0 (PMC7536199; doi:10.1038/s41598-020-73496-0)
Supplement: Supplementary file 1 — Supplementary Figure S1. [file 41598_2020_73496_MOESM1_ESM.pdf]

The effect of slope aspect on vegetation attributes in a mountainous dry valley, Southwest China

Jie Yang[1]*, Yousry A. El-Kassaby[2]*, Wenbin Guan[1]*

[1] School of Ecology and Nature Conservation, Beijing Forestry University, Beijing, China

[2] Department of Forest and Conservation Sciences, Faculty of Forestry, University of British Columbia, Vancouver, BC, Canada

Supplementry material:

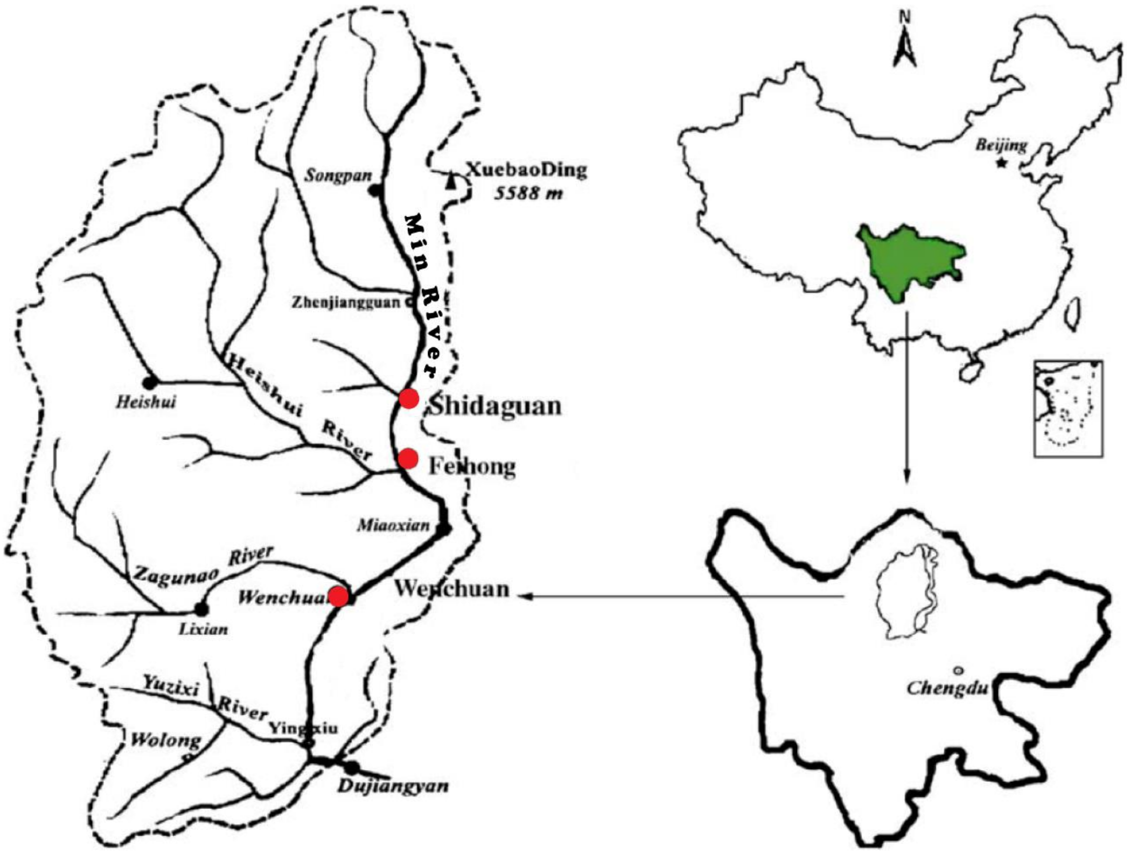

Figure S1. Location of sampling sites along the dry valley in the upper reaches of Min River (modified from Lu *et al.*[1]).

1 Lu, T., Ma, K. M., Zhang, W. H. & Fu, B. J. Differential responses of shrubs and herbs present at the Upper Minjiang River basin (Tibetan Plateau) to several soil variables. *Journal of Arid Environments* **67**, 373-390, doi:10.1016/j.jaridenv.2006.03.011 (2006).
